# Self-Emulsifying Micellization of Crude Extracts from Apple *(Malus domestica* cv. Anna), Plum (*Prunus domestica* cv. Satsuma), and Guava (*Psidium guajava* L.) Fruits

**DOI:** 10.3390/molecules28031297

**Published:** 2023-01-29

**Authors:** Laura A. Calvo-Castro, Andrea Irías-Mata, Daronne Cano-Contreras, Elizabeth Arnáez-Serrano, Randall Chacón-Cerdas, Ricardo Starbird-Pérez, Johan Morales-Sánchez, Carolina Centeno-Cerdas

**Affiliations:** 1 Centro de Investigación en Biotecnología, Escuela de Biología, Instituto Tecnológico de Costa Rica, Cartago P.O. Box 159-7050, Costa Rica; 2 Centro para Investigaciones en Granos y Semillas, Escuela de Agronomía, Universidad de Costa Rica, San José P.O. Box 2060, Costa Rica; 3 Escuela de Química, Instituto Tecnológico de Costa Rica, Cartago P.O. Box 159-7050, Costa Rica

**Keywords:** apple, guava, *Malus domestica* cv. Anna, micelles, plum, polyphenols, *Prunus domestica* cv. Satsuma, *Psidium guajava* L.

## Abstract

Micellar microemulsions are thermodynamically stable self-emulsifying systems that have been used to successfully improve the low oral bioavailability of several bioactive phytochemicals, such as antioxidant polyphenols. However, most studies have reported the micellization of single-compounds or purified chemical fractions; thus, the stability, phytochemical-loading efficiency, and bioactivity of complex crude extracts remain largely unexplored. In this study, we evaluated the effects of micellar emulsification of tropical apple (*Malus domestica* cv. Anna), plum (*Prunus domestica* cv. Satsuma), and guava (*Psidium guajava* L.) extracts regarding particle size and stability, polyphenol-loading efficiency, antioxidant capacity, and cytotoxic activity in human and murine cells. Simple food-grade extraction protocols were implemented to obtain apple, plum, and guava extracts. Total polyphenols, flavonoids, and antioxidant activity (DPPH) were determined in the fruit extracts, and their polyphenol profile was further characterized by liquid chromatography (HPLC-DAD). The dried extracts were mixed into a food-grade, self-emulsifying system, and their cytotoxicity in human and murine cell lines was compared. Our research showed that complex fruit matrixes were successfully emulsified into thermodynamically stable polysorbate-based nanometric micelles with uniform size distribution and consistent pH stability, with potential applications in food and biomedical industries.

## 1. Introduction

Polyphenols are plant secondary metabolites which have at least one aromatic ring with hydroxyl functional groups attached [1]. Long-term intake of polyphenols has been associated with favorable effects against cancer, type-2 diabetes, gastrointestinal problems, and cardiovascular and neurodegenerative diseases, as extensively reviewed in [2,3,4]. Furthermore, polyphenols are generally considered to be a safe dietary element due to their low toxicity in humans [4].

Cultivation under tropical conditions might expose crops to higher solar irradiation which can increase polyphenol accumulation and diversity in plants [5,6]. Accordingly, higher and more diverse polyphenol content and better antioxidant activities have been reported in tropical highland Costa Rican apples (*Malus domestica* cv. Anna, and cv. Jonagold) and plums (*Prunus domestica* cv. Methley, cv. Pisardii and cv. Satsuma) compared with other cultivars from temperate regions [7,8,9]. Moreover, studies have showed the cytotoxic effects of tropical apple and plum extracts against gastric (AGS) and colon (SW-620) human carcinoma cells [8,9] and against breast (MCF7) and lung (NCI-H460) human cancer cells [10].

However, the low bioavailability of polyphenols has been a concern, given that they are limited by problems related to bioaccessibility, solubility, microbial metabolism, and human digestion, absorption, metabolism, and excretion [11,12,13]. Micellar microemulsions are one of the many strategies that have been used to successfully improve the bioavailability of phytochemicals [14,15,16]. Microemulsions are thermodynamically stable self-emulsifying systems which can form spontaneously using simple food-grade ingredients to carry and deliver both hydrophilic and lipophilic substances [17].

Considering the Sustainable Development Goals for 2030 regarding food security and human health, and aiming toward implementing simple food-grade extraction and emulsification protocols that can eventually be translated to small local producers, we compared the polyphenol content and antioxidant capacity of native (powder) and micellar extracts of apple (*Malus domestica* cv. Anna), plum (*Prunus domestica* cv. Satsuma), and guava (*Psidium guajava* L.). Particle size and stability, polyphenol-loading efficiency, and cytotoxic activity on human and murine cell lines were also evaluated. This study confirmed that polysorbate-based self-emulsifying micelles were effective and stable for the micellization of three different crude fruit extracts, which shows the versatility of this emulsion system.

## 2. Results

### 2.1. Phytochemical Analysis

Hydroalcoholic extractions were carried out from freeze-dried fruit materials using different ethanol concentrations. Except for apple peel samples, low ethanol extractions from all fruits showed slightly higher total polyphenol content (TPC) recovery relative to high ethanol concentrations; in contrast, the flavonoid content was numerically higher in all samples when using a high ethanol extraction (Table 1). However, there was no significant difference in the TPC among the fruit extracts, and only the high ethanol guava extract had a significantly superior (*p* < 0.05) total flavonoid content (TFC) compared with the rest (Table 1). Antioxidant activity (DPPH inhibition) was significantly reduced in the whole-apple extracts, but there were no significant differences between high- and low-ethanol extracts in the rest of the samples (Table 1). Based on these results, high ethanol apple peel extract and low ethanol plum and guava extracts were selected to continue with the rest of the experiments. Self-emulsifying micellar formulations were prepared by mixing these selected fruit extracts with a pre-mixed solution of surfactants and oils for a final concentration of 50 mg extract mL^−1^ [18].

**Figure 1:**
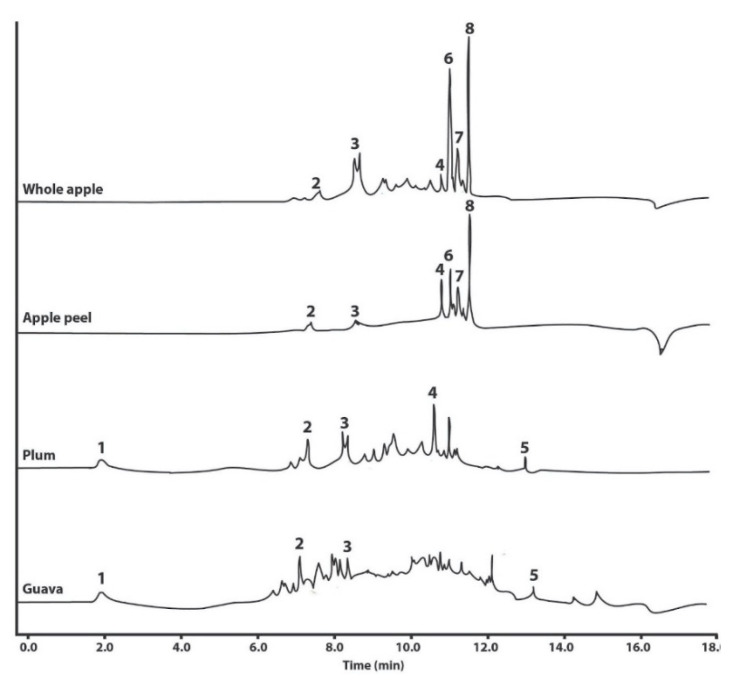
Representative UHPLC-DAD chromatograms of the polyphenols identified in water–ethanol extracts (native powder) of whole apple and apple peel (*M. domestica* cv. Anna), plum (*P. domestica* cv. Satsuma), and guava (*P. guajava* L.). The identity of the polyphenols is as follows: 1, gallic acid; 2, catechin; 3, epicatechin; 4, rutin; 5, quercetin; 6, phloretin xyloglucoside; 7, quercetin glucoside; and 8, phloridzin.

Five polyphenols were detected and quantified in the native extracts as determined by UHPLC-DAD (Table 2). Gallic acid was found in plum and guava extracts, and guava had a significantly three-fold (*p* < 0.05) higher concentration compared with plum. Catechin and epicatechin were identified in all fruits, and guava and plum had significantly superior concentrations (*p* < 0.05) of catechin and epicatechin, respectively, relative to apple. Rutin was detected in apple (in the whole fruit and peel) and plum, with significantly lower content (*p* < 0.05) in whole apple. Quercetin was found in plum and guava, showing two-fold higher (*p* < 0.05) concentrations in guava compared with plum. Three more polyphenols were detected in apple (in the whole fruit and peel) at a high intensity and were tentatively identified based on the UV/Vis spectra in previous reports [19] as phloretin xyloglucoside, quercetin glucoside, and phloridzin (Figure 1). Micellar apple peel extract yielded epicatechin, rutin, phloretin xyloglucoside, quercetin glucoside, and phloridzin in proportions superior to the native extract. The micellar whole plum extract showed higher amounts of the same polyphenols observed in the respective native extract, except rutin. Finally, in the guava formulation, gallic acid was the only identified polyphenol with higher concentrations in the micellar formulation relative to the native extract.

### 2.2. Micellar Characterization

**Figure 2:**
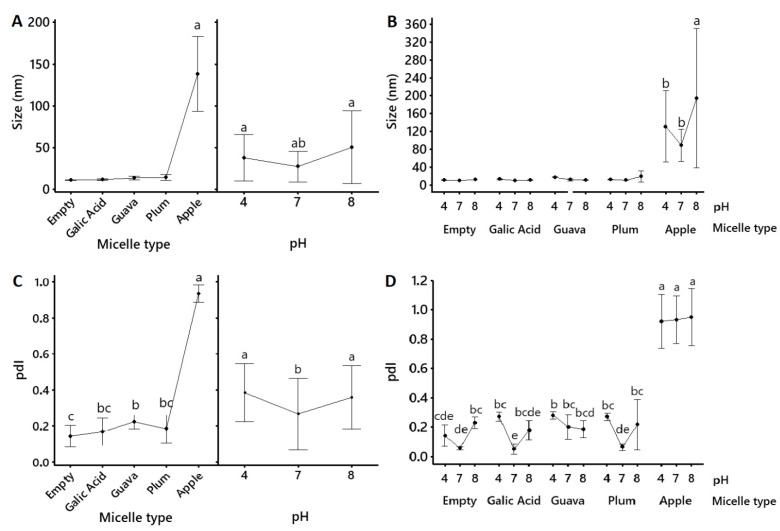
Mean values (mean ± 95% confidence interval, *n* = 6) for the size (nm) (**A**,**B**) and polydispersity index (pdI) (**C**,**D**) of pure gallic acid and apple peel (*M. domestica* cv. Anna), plum (*P. domestica* cv. Satsuma), and guava (*P. guajava* L.) fruit extracts that were solubilized into polysorbate-based micellar formulations. Lines connect mean values. Inserts within (**A**) show the mean size of each micellar formulation (regardless of the pH) on the right and the mean size of all micellar formulations (regardless of the content) on the left. Empty refers to the non-loaded micellar formulation. Statistical differences between the type of micellar fruit extract, the pH, and the type of micellar fruit extract *x* pH were determined using a General Lineal Model, followed by Bonferroni’s multiple comparison post-hoc tests, and they are indicated by superscript letters (*p* < 0.05) (Minitab v.19.1.1., USA).

Emulsification and extract-loading into the micelles was confirmed by a significantly (*p* < 0.05) reduced particle size and polydispersity index (pdI) (Table 3, Figure 2). Particle size also numerically increased in the fruit formulations relative to the non-loaded micelles. The micelles were within the anticipated sizes of 10 to 100 nm [18], and all micellar formulations were more homogeneous (lower size and lower pdI) at pH 7 than at pH 4 or pH 8 (Figure 2). The only exception were the apple peel micelles which had significantly (*p* < 0.05) bigger particles and more heterogeneous sized particles than the other fruit formulations.

**Figure 3:**
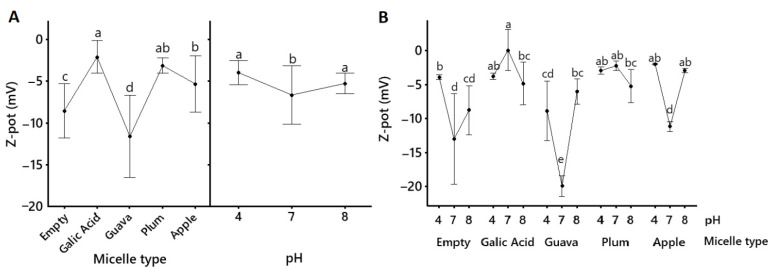
Mean values (mean ± 95% confidence interval, *n* = 6) for the ζ potential (Z-pot) of pure gallic acid and apple peel (*M. domestica* cv. Anna), plum (*P. domestica* cv. Satsuma), and guava (*P. guajava* L.) fruit extracts that were solubilized into polysorbate-based micellar formulations. Lines connect mean values. Empty refers to a non-loaded micellar formulation. Inserts within (**A**) show the mean size of each micellar formulation (regardless of the pH) on the right and the mean size of all micellar formulations (regardless of the content) on the left; (B) shows mean size depending on pH and micelle content. Statistical differences between the type of micellar fruit extract, the pH, and the type of micellar fruit extract *x* pH were determined using a General Lineal Model, followed by Bonferroni’s multiple comparison post-hoc tests, and they are indicated by superscript letters (*p* < 0.05) (Minitab v.19.1.1., USA).

Significantly (*p* < 0.05) increased (closer to zero) ζ-potential in the micellar formulations relative to the native powder (Table 3) suggested a higher tendency of the micelles to aggregate. Surface potential (Figure 3) also showed more negative values at pH 7 for non-loaded, guava, and apple micelles, suggesting more particle repulsion in these formulations at a neutral pH.

### 2.3. Cytotoxicity

**Figure 4:**
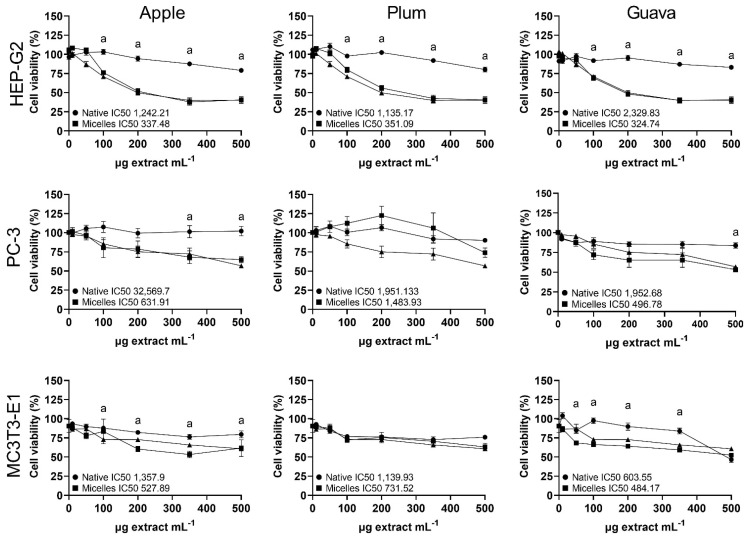
Percentage (mean ± SEM, *n* = 3) cell viability (neutral red assay) in Hep-G2 (human hepatocellular carcinoma), PC-3 (human prostatic adenocarcinoma), and MC3T3-E1 (murine pre-osteoblast fibroblasts) treated for 24 h with native (circled lines) and micellar (squared lines) formulations of apple peel (*Malus domestica* cv. Anna), whole plum (*Prunus domestica* cv. Satsuma), or whole guava (*Psidium guajava* L). Lines with triangles show the effect of equivalent amounts of the empty micelle formulation on the cells. IC_50_ (the concentration of the sample required to inhibit neutral red response to 50% respective to the untreated control) is shown as µg extract mL^−1^. Statistical differences between native and micellar formulations within cell lines were determined by one-way ANOVA, followed by Bonferroni’s multiple comparison post-hoc tests, and they are indicated by superscript letters (*p* < 0.05) (GraphPad Prism v. 9.3.1., USA).

The cytotoxic effect (neutral red assay) of the native and micellar extracts of tropical highland apple, plum, and guava was evaluated in Hep-G2 (human liver cancer cells), PC-3 (human prostate cancer cells), and MC3T3-E1 (murine pre-osteoblast fibroblasts) (Figure 4). Micellar apple peel and guava formulations significantly (*p* < 0.05) increased the cytotoxicity relative to the native powder in all cell lines; meanwhile, the micellar plum formulation had a significantly higher cytotoxicity relative to the native powder only in Hep-G2 cells. However, the fold increase in the cytotoxicity from all the micellar fruit formulations was not significantly different from the cytotoxicity of the non-loaded micelle vehicle solution in all cell lines (Figure 4). The cytotoxicity (IC_50,_ µg empty micellar solution mL^−1^) of the non-loaded micelles was 326.05, 577.98, and 647.13 in the Hep-G2, PC-3, and MC3T3-E1 cells, respectively.

## 3. Materials and Methods

### 3.1. Extract Preparation

Tropical highland apple and plum ripe fruits (*Malus domestica* cv. Anna and *Prunus domestica* cv. Satsuma, respectively) were purchased from local producers in San Marcos, Tarrazú, and San José, Costa Rica (permit R-CM-ITCR-002-2021-OT). Plant identity had been previously confirmed with the support of the Costa Rica National Herbarium [7,8,9]. Whole fruits (without seeds) were water-rinsed, sliced, freeze-dried (Bench Top FDB-8602, OPERON, Gyeonggi, Republic of Korea), grinded (1 mm, IKA® MF10 basic, Milwaukee, USA), and stored at −20 °C until extraction. The powdered material was extracted as previously reported [10] (1:10, plant material to solvent) by three consecutive water–ethanol macerations (15 min incubation in an ultrasonic bath and 45 min of magnetic stirring) at room temperature, followed by solvent elimination in a vacuum evaporator (40 °C, R-300, BUCHI Labortechnik AG, Flawil, Switzerland). The remaining concentrate was freeze-dried and stored at −70 °C. The same protocol was followed using apple peels from the same source and with whole guava fruits (including seeds) (*Psidium guajava* L.) that were purchased from local producers located in Río Grande de Paquera, Puntarenas, Costa Rica (permit R-CM-ITCR-013-2021-OT).

### 3.2. Micellar Formulations

For the micellar formulations, the extracted and dried material was homogenized into a pre-warmed (40 °C) solution of food-grade polysorbate 20 (Sigma-Aldrich, St. Louis, MO, USA), polysorbate 80 (Sigma-Aldrich, St. Louis, MO, USA), and medium chain triglycerides (MCT Oil, Nestlé Health Science, Bridgewater, NJ, USA) for a final concentration of 50 mg extract mL^−1^. This stock solution was later dissolved in aqueous solutions for micellar self-emulsification (1:50) [18].

For each sample, 1:50 micelle dilutions were made in distilled water and sonicated for 60 s (40 kHz) in an ultrasonic bath (CPI08895-21, Cole-Palmer, Vernon Hills, IL, USA). Then, 800 µL of the sample was poured into a disposable capillary cell (DTS0012 and DTS1070) designed for a Zetasizer Nano (Nano ZS, Malvern-Panalytical, UK). The hydrodynamic diameter and the polydispersity index (pdI) of the particles were measured using the corresponding diffraction index and the viscosity of the dispersant to quantify the sizes and their abundance. The ζ-potential was determined by measuring the potential of the particles of the particles at the slipping plane using the principle of electrophoretic mobility. Measurements were made at three different pH values (4, 7, and 8). The same procedure was repeated with 1 mg ml^−1^ water dilutions of the native extracts. A GLM (General Linear Model) type design was used to identify possible interactions between the type of micelle and the pH on the characteristics of size, polydispersity, and surface charge. 

### 3.3. Phytochemical Analysis

Total polyphenol content (TPC) was determined as described by Rojas-Garbanzo et al. [20]. Briefly, native fruit extracts and micellar formulations (10 mg extract mL^−1^ in 70% ethanol) were incubated with Folin–Ciocalteu reagent and sodium carbonate solution (75 g L^−1^) at 50 °C for 15 min. The absorbance was registered at λ = 620 nm (FLUOstar OPTIMA, BMG LABTECH, Ortenberg, Germany), and TPC was calculated by comparing it to an external calibration curve of gallic acid (10–80 mg GAE L^−1^, *r*^2^ = 0.9923). All samples were evaluated in triplicates, and the results are expressed as mg of gallic acid (>99% purity, Sigma-Aldrich, St. Louis, MO, USA) equivalents (GAE) per gram of freeze-dried extract (DW). TPC is shown as the mean ± standard deviation.

Total flavonoid content (TFC) was analyzed via the aluminum chloride method as described by Fernandes et al. [21]. Samples (10 mg extract mL^−1^ in methanol) were mixed with 2% aluminum chloride solution (in methanol) and incubated at room temperature for 10 min. The absorbance was measured at λ = 450 nm (FLUOstar OPTIMA, BMG LABTECH), and TFC was calculated by comparing it to an external calibration curve of quercetin (0–160 mg QE L^−1^, *r^2^* = 0.9814). All samples were tested in triplicates, and the results are expressed as mg of quercetin (>99% purity, Sigma-Aldrich, St. Louis, MO, USA) equivalents (QE) per gram of freeze-dried extract (DW). TFC is shown as the mean ± standard deviation.

The polyphenols profile was determined using UHPLC-DAD based on a previously reported protocol by Fratianni et al. [22] with slight modifications. Aliquots of 5 μL of each sample (10 mg native extract mL^−1^ in 70% ethanol and 1 mg micellar formulation mL^−1^ in H_2_O) were injected into an ultra-high performance liquid chromatography (UHPLC) that was coupled with a diode array detector (Ultimate 3000 TSQ Endura, serie TQH-E1-0288, Thermo Fisher Scientific, Waltham, MA, USA). Polyphenols were separated on a Acquity UPLC CSH C18 column (1.7 μm particle size, 100 × 2.1 mm) maintained at 30 °C using an acetic acid aqueous solution (7.5 mM; eluent A) and acetonitrile (eluent B) at a flow rate of 0.25 mL min^−1^. The elution gradient was as follows: isocratic 95% A for 0.8 min, from 95% to 80% A in 5.2 min, isocratic 80% A for 0.5 min, from 80 to 70% A in 1 min, isocratic 70% A for 0.2 min, from 70 to 50% A in 2.3 min, from 50 to 0% A in 1 min, isocratic 0% A for 2.5 min, and then back to 95% A in 0.1 min while running isocratic at 95% A until 18 min total run. Peaks were recorded and integrated using Chromeleon software (version 7.0, Thermofisher Scientific) and identified and quantified using UV-Vis wavelengths and authentic polyphenols standards (Sigma-Aldrich, St. Louis, MO, USA).

### 3.4. Antioxidant Activity (DPPH Assay)

Antioxidant activity was determined using the DPPH radical scavenging assay as described by Wang et al. [23]. Samples (10 mg extract mL^−1^ in 70% ethanol) were diluted in methanol in increasing concentrations and mixed with 0.5 mM DPPH solution and incubated at 37 ºC for 30 min in the dark. The absorbance was measured at λ = 544 nm (FLUOstar OPTIMA, BMG LABTECH), and the radical scavenging activity was calculated as DPPH percentage inhibition = [Ac − (As − Ab)]/(Ac × 100), with Ac, As, and Ab as the absorbance values corresponding to the negative control (DPPH solution without the sample), the sample, and the blank (the sample without DPPH), respectively. Data were plotted as DPPH percentage inhibition versus sample concentration, and IC_50_ (the concentration of the sample required to inhibit DPPH response to 50% respective to the untreated control) was calculated from the linear equation of each curve (Graph-Pad Prism, v. 9.3.1, GraphPad Software, San Diego, CA, USA).

### 3.5. Cell Culture

The cell lines used in this study were Hep-G2 (ATCC HB-8065™ human hepatocellular carcinoma, passages 13 to 20), PC-3 (ATCC CRL-1435™, human prostatic adenocarcinoma, passages 2 to 8), and MC3T3-E1 (ATCC HTB-22^™^, murine pre-osteoblast fibroblasts, passages 19 to 20). Hep-G2 cells were cultured in DMEM (4.5 g L^−1^ glucose, GIBCO, Grand Island, NY, USA), while PC-3 cells were cultured in RPMI (GIBCO), and MC3T3-E1 cells were cultured in α-MEM (GIBCO). All media were supplemented with 10% fetal bovine serum (FBS, Sigma-Aldrich, St. Louis, MO, USA), 2% L-glutamine (4 mM, GIBCO), 1% sodium pyruvate (0.11 mg mL^−1^, Sigma-Aldrich, St. Louis, MO, USA), and 1% antibiotics (1 × 10^4^ IU mL^−1^ penicillin and 1 × 10^4^ µg mL^−1^ streptomycin, GIBCO). All cell lines were maintained at 95% humidity and 5% CO_2_ at 37 °C.

### 3.6. Cell Viability

Cells lines were seeded onto 96-well plates (1–5 × 10^5^ cells cm^−2^) and treated for 24 h with increasing concentrations (10–500 mg extract mL^−1^) of the native and micellar fruit extracts diluted in the respective culture medium. Cell viability was measured by Neutral Red assay (4 mg mL^−1^; Invitrogen, Waltham, MA, USA) as described by Repetto et al. [24]. Supravital dye incorporated into the lysosomes was measured at λ = 544 nm (FLUOstar OPTIMA, BMG LABTECH, Ortenberg, Germany). Cell viability was normalized as relative percentages in comparison to untreated controls. A linear dispersion curve of cell concentration vs. percentage viability was calculated, from which the half-maximal inhibitory concentration (IC_50_) of each extract was determined. The effect of the “empty micelles” (micelle vehicle solution diluted 1:50 in water without extract) in cell viability was also calculated. Data are shown as the mean ± standard error of the mean (*n* = 3).

### 3.7. Statistical Analysis

Initial comparisons for size, polydispersity, and surface charge between native extract and micelles were performed using a two-sample *t*-test or one-way ANOVA followed by Tukey’s multiple comparison test (apple) which aimed to evidence structuration process. Next, the characterizations of size, polydispersity, and surface charge of the micelles were evaluated using a GLM (General Linear Model) design followed by Bonferroni’s multiple comparisons post-hoc test using the principle of interaction of pH and micelle type. The statistical assumptions of normality and homoscedasticity were evaluated using the corresponding Anderson–Darling and Levene tests using the software Minitab 19 (v.19.1.1.; Minitab Inc, State College, PA, USA). For the phytochemical characterization (TPC, TFP, DPPH) and cell culture assays, normality was tested via the Kolmogorov–Smirnov and D’Agostino–Pearson tests. TPC, TFP, and DPPH inhibition and differences between native and micellar treatments within cell lines were compared between fruit extracts by one-way ANOVA, followed by a Bonferroni’s multiple comparison post-hoc test using the software package GraphPad Prism (v. 9.3.1.; GraphPad Software, USA). Polyphenol concentrations determined by HPLC were compared by one-way ANOVA, followed by Tukey’s multiple comparison test using Rstudio (4.1.0., USA). A confidence level of 95% and α = 0.05 was set for all the statistical analysis.

## 4. Discussion

In this study, simple food-grade extraction protocols were implemented to obtain apple (*M. domestica* cv. Anna), plum (*P. domestica* cv. Satsuma), and guava (*P. guajava* L.) extracts with quantifiable antioxidant phenolic content, which were successfully solubilized into micellar microemulsions that exhibited equivalent cytotoxic effects relative to the native powder in human and in murine cell lines.

Regarding the phytochemical profile of the extracts, total phenolic content (TPC), total flavonoid content (TFC), and antioxidant capacity (DPPH inhibition) were compared between high- and low-ethanol whole fruit extracts. Apple peel (exocarp) was also included based on previous reports [7,9]. Apple and plum seeds were removed as they are not commonly ingested and because of their amygdalin content which can produce cyanide toxicity [25]. Given the few differences in the polyphenol content and the antioxidant activity between high- and low-ethanol extracts (Table 1), and considering affordability, low ethanol plum and guava extracts were selected to continue with the cytotoxicity experiments. Apple peel was chosen over whole apple based on the same parameters, but high ethanol was selected due to its superior polyphenol content (Table 1 and Table 2, Figure 1).

There were no significant differences in the TPC, TFC, and antioxidant activity among the chosen fruit extracts (Table 1), which suggests a similar amount of total polyphenols; however, gallic acid and quercetin were absent in apple peel; rutin was absent in guava; guava had significantly higher gallic acid, catechin, and quercetin content; and plum had significantly higher epicatechin content (Table 2). Of note, as shown in the chromatograms (Figure 1), there are plenty of other compounds (possibly some other polyphenols) in all the extracts that were not identified and quantified due to limitations of the DAD detector and a lack of authentic standards for comparison; thus, they can partially explain the contradictions among the TPC, TFC, and antioxidant activity results against the reported polyphenol profiles. Nevertheless, the identified polyphenols in the fruit extracts agree with previous publications: catechin, epicatechin, rutin, phloretin xyloglucoside, quercetin glucoside, and phloridzin were reported in apples by Tsao et al. [19] and Navarro et al. [7], with phloridzin as the most predominant. Meanwhile, gallic acid, catechin, epicatechin, rutin, and quercetin have also been previously reported in guava [26] and plum [7,27].

Emulsification and extract-loading into the micelles was confirmed by significantly (*p* < 0.05) smaller and more heterogenous particle size distributions (pdI) in the micellar formulations relative to the native powder, along with changes in their ζ-potential (Table 3, Figure 2 and Figure 3). Size and polydispersity index may predict the transport of nanoparticles through biological systems. The smaller the particles, the more versatile they are [28]. Surface potential relates to the interaction of the particles with biological membranes [29] and, in combination with particle size, it defines variable cellular uptake and biodistribution [30]. Moreover, ζ-potential provides information regarding the stability of the ionic colloidal system. When it is close to zero, the particles tend to attract each other, and flocculation occurs. ζ-potential magnitudes higher than 30 to −30 mV are usually considered stable ionic systems [31]. Nonionic surfactants are stabilized mainly due to steric hindrance [31]. Stability is often influenced by the surfactant due to its chemical potential, which affects how surface–surface interactions become repulsive and enhance dispersion [32].

Our research confirmed that the obtained nanometric micelles were mostly within the anticipated sizes (Table 3, Figure 2) of 10 to 100 nm [18], with expected variations in size, polydispersity, and surface potential (Table 3, Figure 2 and Figure 3), possibly due to the different compositions of the fruit extracts [33]. The particle sizes (Table 3, Figure 2) of the gallic acid, guava, and plum micelles (<40 nm) remained statistically the same (*p* > 0.05) as the non-loaded micellar formulation, with a small numerical increase in the guava and plum micelles, which might account for micelle-loading. Micelles were also tested at acidic, neutral, and slightly alkaline pH to account for similar biological conditions in the gastrointestinal tract. The particle sizes of gallic acid, guava, and plum micelles were not significantly affected by the pH, but acid and alkaline conditions caused greater polydispersity relative to pH 7 (Figure 2). All loaded polysorbate-based micelles showed negative ζ-potential values; however, surface potential at pH 4 and pH 8 was significantly closer to zero relative to pH 7, which indicates that neutral pH was the best condition to avoid micellar agglutination (Table 3, Figure 3). However, all micellar formulations remained visibly stable in our laboratory at 4 °C for over 12 months as clear yellow viscous solutions with no precipitates (data not shown). Similar methods were used in other investigations that coincide with the ability of the micellar interaction system to encapsulate functional compounds, such as antioxidants [34]. Moreover, very similar formulations have been shown to significantly enhance the bioavailability of curcumin [35,36] and resveratrol [37] in humans. Thus, given that this micellar emulsion is nonionic, its well-known stability might be supported by an alternative electrostatic repulsion mechanism, which is of interest for future investigations.

Apple peel micelles were the exception as they showed significantly (*p* < 0.05) bigger sizes (reaching up to 138 nm) and much higher size-heterogeneity and pH instability than the other micellar formulations. This behavior could be related to the higher polyphenol diversity in the apple peel extract, which showed greater flavonoid content (Table 1) and the exclusive presence of phloretin xyloglucoside, quercetin glucoside, and phloridzin (Table 2, Figure 1). Further testing will be required to determine how specific polyphenol content influences micellar behavior. Moreover, all extracts were crude preparations, comprising complex mixes where several other phytochemicals may also be included, which might also influence micellization and stability. However, these results indicate that the obtained polysorbate-based formulations were efficient for solubilizing all three fruit extracts, which produced mostly nanometric micelles with a generally stable size distribution.

Regarding micelle-loading, the micellar formulations exhibited different phenolic proportions compared with the native dry extracts (Table 2). Certain compounds could have been lost due to degradation, precipitation, insolubility, or chemical instability, or they could have become too diluted in the micelles to be detected. Compounds with increased concentrations in the micellar formulations could be the result of improved stability and/or solubility, but further testing is required to explore this effect. A similar behavior was reported after the encapsulation of 6-prenylnaringenin in similar micellar formulations, which showed up to 65% higher concentrations compared with the native formulation at presumably equivalent amounts [38].

Finally, the native extracts exhibited half-maximal inhibitory concentrations against human and murine cells (neutral red cell viability assay) (Figure 4) within the range considered weakly active (IC_50_ 100–1000 μg mL^−1^) or inactive (IC_50_ > 1000 μg mL^−1^) [39]. In contrast, Navarro et al. [8,9] had previously shown moderately active (IC_50_ 20–100 μg mL^−1^) cytotoxic effects from apple peel and plum flesh against gastric and colon carcinoma cells. Such differences in polyphenol content and bioactivity between studies might be due to diverse climates, orchard practices, ripeness, storage conditions, extraction methods, and analytical techniques. Furthermore, Navarro et al. [8,9] used more purified polyphenol fractions, while we used complex extracts with lower polyphenol contents. Nonetheless, this suggests that our native fruit formulations were weakly active to inactive in the tested cell lines, while they retained measurable TPC content and antioxidant activity, which would be appropriate for developing future oral or topical applications. Considering that the increased delivery of xenobiotics has raised concerns regarding dose-related and vehicle-induced negative effects, these results suggest that micellar emulsification did not increase the extract´s cytotoxicity, and they may provide better possibilities for the effective delivery of bioactive phytochemicals in relevant concentrations to diverse biological systems. However, the possible cytotoxicity of the micellar vehicle itself warrants further studies (Figure 4), and the bioactivity of the micellar formulations should also be tested in authentic normal human cells.

Of note, the relevant effects of pH on the polydispersity and surface potential of the micelles highlight the relevance of this factor when considering the stability of these formulations in the digestive tract where acidic pH variations in the stomach to more alkaline conditions in the intestines have a relevant impact on the release and absorption of cargo compounds [40]. Given that very similar micellar formulations have already shown increased oral bioavailability of other phytochemicals in humans [35,36,37], further experiments are required to elucidate the transport mechanisms of these micellar systems in diverse administration pathways.

## 5. Conclusions

Crude extracts of three different tropical fruits (apple, plum, and guava) were successfully emulsified using a thermodynamically stable polysorbate-based micelle system, which was previously known to improve the oral bioavailability of other bioactive phytochemicals. The obtained nanometric micelles showed steady size distributions, pH stability, and ζ-potential values that may provide alternative biological responses. Our research provides information on the stability, phytochemical-loading efficiency, and bioactivity of tropical fruit crude extracts. It shows that complex matrixes can be successfully solubilized into edible, self-emulsifying micelles, with potential applications in food and biomedical industries. Further testing will be required to determine how specific polyphenol content influences micellar behavior stability. Future research should also consider the release and absorption of cargo content through the gastrointestinal system and other forms of administration as well as more detailed bioactivity experiments both in vitro and in vivo.

## Figures and Tables

**Table 1:** Total polyphenol content (TPC), total flavonoid content (TFC), and relative percentage DPPH inhibition of apple (*M. domestica* cv. Anna), plum (*P. domestica* cv. Satsuma), and guava (*P. guajava* L.) extracts (mean ± SD, *n* = 6).

Extract	TPC (µg GAE mg^−1^ Extract)	TFC (µg QE mg^−1^ Extract)	DPPH (IC_50_) (µg Extract mL^−1^)
Whole Apple (*M. domestica* cv. Anna)			
High ethanol	15.70 ± 3.99 ^a^	1.09 ± 0.25 ^a^	55.66 ± 19.33 ^a^
Low ethanol	22.23 ± 12.66 ^a^	0.32 ± 0.27 ^a^	30.26 ± 0.23 ^b^
Apple Peel (*M. domestica* cv. Anna)			
High ethanol	25.72 ± 5.99 ^a^	2.93 ± 1.84 ^a^	19.06 ± 5.52 ^b^
Low ethanol	23.53 ± 11.45 ^a^	1.11 ± 0.16 ^a^	25.43 ± 26.38 ^b^
Whole Plum (*P. domestica* cv. Satsuma)			
High ethanol	20.29 ± 4.68 ^a^	1.84 ± 0.77 ^a^	15.84 ± 1.62 ^b^
Low ethanol	23.45 ± 3.87 ^a^	0.18 ± 0.44 ^a^	15.39 ± 2.35 ^b^
Guava (*P. guajava* L.)			
High ethanol	15.17 ± 3.20 ^a^	5.85 ± 1.12 ^b^	14.28 ± 3.99 ^b^
Low ethanol	24.27 ± 5.19 ^a^	1.46 ± 0.44 ^a^	16.04 ± 5.87 ^b^

IC_50_, concentration of the sample required to inhibit DPPH response to 50% respective to the untreated control; GAE, gallic acid equivalents; QE, quercetin equivalents. One-way ANOVA followed by a Bonferroni post-hoc test was carried out within each column. Rows within columns that do not share the same superscript letters are significantly different at *p* < 0.05 (GraphPad Prism v. 9.3.1., USA).

**Table 2:** Specific polyphenols (mean ± SD; *n* = 3) detected and quantified by HPLC-DAD in water–ethanol extracts (native powder and their micellarized formulations) of apple (*M. domestica* cv. Anna), plum (*P. domestica* cv. Satsuma), and guava (*P. guajava* L.) fruits.

Compound	Retention Time (min)	UV/Vis Wavelength (nm)	Polyphenol Concentration [ug mg^−1^ Extract] in Native Extracts				Proportion of Polyphenols in Micelles/Native Extract		
			Whole Apple	Apple Peel	Plum	Guava	Apple Peel	Plum	Guava
Gallic acid	2.0	201/220/270	n.d.	n.d.	0.81 ± 0.49 ^a^	2.65 ± 0.10 ^b^	n.d.	19.29	4.97
Catechin	7.7	201/278	0.83 ± 0.49 ^a^	1.27 ± 0.42 ^a^	9.44 ± 6.87 ^a^	24.07 ± 0.88 ^b^	n.d.	2.91	n.d.
Epicatechin	9.0	201/281/324	2.68 ± 1.34 ^a^	2.91 ± 0.36 ^a^	7.05 ± 1.25 ^b^	2.26 ± 0.08 ^a^	2.56	1.09	n.d.
Rutin	11.2	201/256/355	1.08 ± 0.07 ^a^	5.54 ± 0.79 ^b^	5.97 ± 1.21 ^b^	n.d.	1.14	0.45	n.d.
Quercetin	13.1	201/255/370	n.d.	n.d.	0.55 ± 0.30 ^a^	1.09 ± 0.04 ^b^	n.d.	17.26	n.d.
Phloretin xyloglucoside ^ti^	11.4	191/220/286	n.c.	n.c.	n.d.	n.d.	0.94	n.d.	n.d.
Quercetin glucoside ^ti^	11.6	199/257/353	n.c.	n.c.	n.d.	n.d.	1.01	n.d.	n.d.
Phloridzin ^ti^	11.9	191/221/286	n.c.	n.c.	n.d.	n.d.	1.28	n.d.	n.d.

n.d., not detected; n.c., not quantified; and ti, tentatively identified polyphenols based on Tsao et al. [19]. The relative amounts of tentatively identified polyphenols in micelles relative to the native formulation were estimated by comparing the peak area. The other polyphenols were identified based on their concentration as determined by comparison with authentic standards. For polyphenol concentrations, one-way ANOVA followed by Tukey’s multiple comparison tests were carried out within each row. Data within rows that do not share the same superscript letters are significantly different at *p* < 0.05 (Rstudio 4.1.0., Boston, MA, USA).

**Table 3:** Particle size, polydispersity index (pdI), and ζ-potential (*n* = 6) of apple (*M. domestica* cv. Anna), plum (*P. domestica* cv. Satsuma), and guava (*P. guajava* L.) fruit extracts as native powder and their micellarized formulations.

Sample	Formulation	Size (nm)	pdI	ζ–Potentials (mV)
Gallic Acid				
Commercial standard	Native	156.93 ± 4.51 ^a^	0.239 ± 0.017 ^a^	−15.27 ± 8.59 ^b^
	Micelles	10.56 ± 0.04 ^b^	0.054 ± 0.014 ^b^	0.04 ± 2.87 ^a^
Apple (*M. domestica* cv. Anna)				
Whole fruit	Native	228.30 ± 28.50 ^a^	0.361 ± 0.048 ^b^	−13.87 ± 3.91 ^a^
Apple peel	Native	244.60 ± 12.81 ^a^	0.313 ± 0.008 ^b^	−24.28 ± 2.95 ^a^
	Micelles	138.18 ± 6.19 ^b^	0.935 ± 0.014 ^a^	−5.36 ± 0.48 ^b^
Plum (*P. domestica cv. Satsuma*)				
Whole fruit	Native	139.53 ± 1.33 ^a^	0.189 ± 0.019 ^a^	−11.43 ± 1.47 ^b^
	Micelles	10.68 ± 0.03 ^b^	0.066 ± 0.009 ^b^	−2.25 ± 0.66 ^a^
Guava (*P. guajava* L.)				
Whole fruit	Native	245.63 ± 15.07 ^a^	0.265 ± 0.025 ^a^	−53.17 ± 0.68 ^b^
	Micelles	11.89 ± 1.02 ^b^	0.202 ± 0.033 ^a^	−19.90 ± 0.60 ^a^

Statistical differences between native vs. micellar formulations within each sample were determined by a two-sample *t*-test or one-way ANOVA and Tukey´s multiple comparison test (apple), and they are indicated by superscript letters (*p* < 0.05) (Minitab v.19.1.1., USA).

## Data Availability

The datasets from the current study are available from the corresponding author on reasonable request.
